# Mother-to-child transmission of HIV: the pre-rapid advice experience of the university of Nigeria teaching hospital Ituku/Ozalla, Enugu, South-east Nigeria

**DOI:** 10.1186/1756-0500-5-305

**Published:** 2012-06-19

**Authors:** Ngozi S Ibeziako, Agozie C Ubesie, Ifeoma J Emodi, Adaeze C Ayuk, Kene K Iloh, Anthony N Ikefuna

**Affiliations:** 1 Department of Pediatrics, Faculty of Medical Sciences, University of Nigeria, Enugu, Nigeria; 2 Department of Pediatrics, University of Nigeria Teaching Hospital, Ituku/Ozalla, Enugu, Nigeria

**Keywords:** HIV, Mother-to-child transmission, Risk factors

## Abstract

**Background:**

Mother-to-child transmission of human immune deficiency virus (HIV) is the most common route of HIV transmission in the pediatric age group. A number of risk factors contribute to the rate of this transmission. Such risk factors include advance maternal HIV disease, lack of anti-viral prophylaxis in the mother and child, mixing of maternal and infant blood during delivery and breastfeeding. This study aims to determine the cumulative HIV infection rate by 18 months and the associated risk factors at the University of Nigeria Teaching Hospital, Enugu.

**Results:**

A retrospective study, involving HIV exposed infants seen at the pediatric HIV clinic of UNTH between March 2006 and September 2008. Relevant data were retrieved from their medical records. The overall rate of mother to child transmission of HIV in this study was 3.9% (95% CI 1.1%- 6.7%). However, in children breastfed for 3 months or less, the rate of transmission was 10% (95% CI −2.5%-22.5%), compared to 3.5% (95% CI 0.5%-6.5%) in children that had exclusive replacement feeding.

**Conclusions:**

This retrospective observational study shows a 3.9% cumulative rate of mother-to-child transmission of HIV by 18 months of age in Enugu. Holistic but cost effective preventive interventions help in reducing the rate of mother-to-child transmission of HIV even in economically-developing settings like Nigeria.

## Background

The first documented case of Acquired Immune Deficiency Syndrome (AIDS) in Nigeria was in 1986 in a 13 year old child in Calabar, Cross River State [1]. Since then, children have continued to remain vulnerable to this epidemic in Nigeria. Children can be infected with the virus through mother-to-child transmission (MTCT), blood transfusion, unprotected sex and through the use of non-sterile sharp objects [1,2]. MTCT is the most common route and is responsible for as much as 70 to 95% [3-6] of the infection in the pediatric age group. The next most common route of HIV transmission in children living in economically-developing countries is blood transfusion.[7,8]. This route accounts for about 5 to 20% of pediatric AIDS [3,4].

MTCT can occur in utero, during labor and delivery, and postnatally through breastfeeding. A number of risk factors for MTCT of HIV have been documented. The risk factors associated with transmission during labor are prolonged rupture of uterine membrane for more than 4 hours, prolonged labor, mixing of maternal and fetal blood which happens more with tears and episiotomies [5,9]. The risk factors associated with transmission post-natally are breastfeeding and mixed feeding [5,9]. The World Health Organization (WHO) had noted that with successful interventions, the risk of MTCT can be reduced to less than 2% [10]. The effectiveness of antiretroviral drugs (ARVs) in preventing MTCT has been demonstrated especially during the pre-natal and intra-partum periods [7,8]. These ARVs can be given as prophylaxis or highly active antiretroviral therapy to the mother and as prophylaxis to the baby.

Infant feeding in the context of HIV is still a challenging issue in economically-developing countries; with breastfeeding contributing about 30 to 50% of the MTCT of HIV [8]. Breast milk is usually readily available, free of charge and contains protective agents such as phagocytes, lactoferrin, oligisacharides and immunoglobulins [11]. These protective agents help protect against common childhood illnesses such as diarrhea and respiratory infections [11]. Unfortunately, breastfeeding is also a mode of HIV transmission. In economically-developed countries like the United States and United Kingdom, the advice from national health agencies is usually to avoid breastfeeding altogether because the risk of HIV transmission far outweighs the risks associated with replacement feeding [12]. ^*.*^ In economically developing countries on the other hand, the recommendation for infant feeding is less emphatic, and initially depended on a mother's individual situation and choice. The WHO had posited that “when replacement feeding is acceptable, feasible, affordable, sustainable and safe, avoidance of all breastfeeding by HIV-infected mothers is recommended; otherwise, exclusive breastfeeding is recommended during the first months of life”[13]. It may not be surprising then that in Africa, between one third and one half of infant HIV infections are due to breastfeeding [14]. Currently, WHO in its rapid advice of 2009 recommends that HIV exposed infants in economically developing countries should be breast fed for up to a year with a proviso; atleast either the baby or mother remains on antiretroviral drug(s) [15]. This is a retrospective observational study of the pre-Rapid Advice era at University of Nigeria Teaching Hospital (UNTH). It is hoped that our findings will provide the basis for comparison with the rate of transmission after implementation of the new guideline on HIV and infant feeding.

## Results

**Figure 1:**
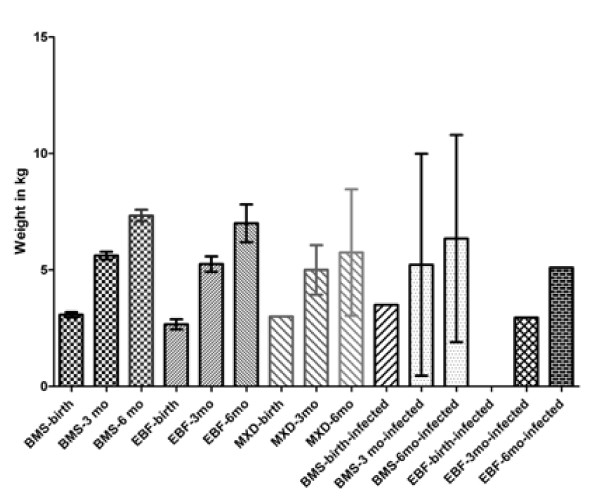
This bar plot shows the average infant weight data for the 171 uninfected and 7 infected children with error bars (from the minimum to the maximum value) at birth, 3rd and 6th month grouped into the feeding choices made.

A total of 227 HIV exposed babies were enrolled into the HIV Exposed Infant Clinic and complete data retrieved for 178 babies. The forty-nine children for whom the data were not retrieved were lost to follow up. These were children that did not return to the clinic after their first visit and were not analyzed further. Out of the 178, there were 101 males and 77 females with male: female ratio of 1.3:1 as shown in Table 1. This difference in gender was not statistically significant (*χ*^2^ = 3.24, p = 0.72). Data on ante-natal history and place of delivery were available in 128 subjects; 24.2% of the mothers had ante-natal care at UNTH but delivered elsewhere, 0.4% delivered at UNTH but had ANC elsewhere, 40.6% had both ANC and delivery at UNTH while 31.3% had neither ANC nor delivery at UNTH. There was a statistically significant differences in the rate of HIV transmission between the children depending on places of ANC and delivery (*χ*^2^ = 37.31, p < 0.05). The average birth weight of all babies seen was 3.2 ± 0.6 kg. The average weight at 3 and 6 months respectively for the infected children and those that had mixed feeding were lower compared to those that received breast milk substitutes or exclusive breastfeeding as depicted in Figure 1. However, a Kruskal Wallis test on one way ANOVA showed no statistically significant difference amongst the three modes of feeding in respect to the average weights of the children.

**Table 1:** Demographic characteristics and feeding choices for the 178 children in whom HIV infection was ascertained by 18 months

**Feeding**	**Male**	**Female**	**Total**	**HIV infected**
Choice	**(%)**	**(%)**	**(%)**	
BMS	**80(44.9)**	**62 (34.9)**	**142 (79.8)**	5
EBF (1)	**11 (6.2)**	**9 (5.0)**	**20 (11.2)**	2
EBF (2)	1(0.6)	**1(0.6)**	**2 (1.1)**	0
Mixed feeding	**9 (5.1)**	**5 (2.8)**	**14 (7.9)**	0
Total	**101(56.7)**	**77 (43.3)**	**178 (100)**	7

BMS = Breast Milk Substitute; EBF (1) = Exclusive Breastfeeding for 3 months or less; EBF (2) = Exclusive Breastfeeding for 3–6 months. There was no statistically significant difference in the risk of becoming HIV infected between children in different categories at 95% confidence level.

Legend: The table is showing the demographic characteristics and feeding choices for the 178 children in whom HIV-infection status was ascertained by 18 months.

Concerning the 178 babies whose data were retrieved, the partners of 82 (46.1%) mothers were also HIV sero-positive, 64 (36%) had discordant results with their partners (sero-negative spouses) while the sero-status of the partners of 32 (18%) women were not recorded. Most of the women (82.6%) received anti-retroviral drugs during the index pregnancy. Delivery of the babies was through the vaginal route in 87.1% of the mothers, caesarean operation in 12.4% while the mode of delivery could not be ascertained in one woman. None of the caesarean deliveries was performed to prevent MTCT of HIV. Indications for caesarean operations varied from poor progress of labor to previous scars. Majority (82.6%) of the infants received single dose nevirapine (SdNVP) at birth that was followed with daily Ziduvudine for 6 weeks. The rest received Zidovudine only (7.9%), SdNVP only (1.1%) and no anti-retroviral prophylaxis in 8.4% of the children. All the anti-retroviral medications were prescribed at UNTH Enugu.

The Infant feeding options available at UNTH Enugu for HIV positive mothers were exclusive breast feeding for short duration (3 to 6 months) or exclusive replacement feeding using breast milk substitutes. One hundred and forty-two babies (79.8%) were on exclusive breast milk substitute for 3 months, 22 (12.3%) received only breast milk for less than 6 months while 14 (7.9%) had mixed feeding (breast feeding and breast milk substitute) as shown in Table 1. None of the mothers breastfed for more than 6 months. Seven of the babies in this study were confirmed HIV positive while 171 were negative, giving an overall MTCT of HIV rate of 3.9% (95% CI 1.1%- 6.7%). Of the 7 infected babies, 3 were males and 4 females. Except for one, all the mothers of the HIV positive infants received antiretroviral medications during pregnancy at UNTH Enugu. Assessment of the mothers’ adherence to the ARVs was by verbal questioning. There was no statistically significant difference in infants’ HIV sero-status with respect to anti-retroviral medications among the mothers during pregnancy (*χ*^2^ = 0. 824, p = 0.05). All of the 7 fathers of these HIV positive babies were also HIV sero-positive. Six of the 7 HIV positive babies were delivered via the vaginal route compared to one by caesarean section. There was no statistically significant difference between the two routes of delivery and Infants’ HIV status (*χ*^2^ = 0. 968, p = 0.64). None of the 7 HIV infected babies was delivered at UNTH; one was actually delivered at home by a traditional birth attendant.

In terms of infants’ post exposure prophylaxis, 6 infants (4.1%) among those that received both single dose nevirapine and 6 weeks of zidovudine became HIV infected compared to only child (6.7%) from the group that received no post exposure prophylaxis. This difference was not statistically significant (*χ*^2^ = 0.96, p = 0.811).

Among babies that had exclusive replacement feeding, 3.5% (95% CI 0.5%-6.5%) became HIV positive compared to 10% (95% CI −2.5%-22.5%) among those that had exclusive breast feeding for 3 months or less. Interestingly, none of the babies that were breastfed exclusively for more than 3 months or had mixed feeding tested HIV positive. However, there was no statistically significant difference between the various modes of infant feeding and infant’s HIV sero-positivity (*χ*^2^ = 2.67, p = 0.446).

## Discussion

Eighteen percent of the children were lost to follow up. They did not return to the clinic after the initial enrolment and we are not sure what happened to them since our program does not have any mechanism in place for tracking defaulters. The cumulative mother-to-child HIV transmission rate in this study was 3.9% (95% CI 1.1%- 6.7%). In the absence of any efficacious intervention to prevent transmission, the risk of mother-to-child transmission of HIV is approximately 15 to 42% [16]. In economically-developed countries, perinatal transmission of HIV has been reduced to 1% as a result of the use of Pediatric AIDS Clinical Trials Group study (PACTG 076) regimen, highly active anti-retroviral therapy (HAART), appropriate management of labor and delivery; and avoidance of breastfeeding [17]. In a UK study, the rate of MTCT of HIV was 2% [18], comparable to less than 2% reported from clinical trials in other developed countries [19]. The UK study further noted that 70.6% of their subjects received HAART consisting of 3 or more drugs [18].

In economically-developing countries, the mother-to-child HIV transmission rate varies in the various literatures depending on the available interventions in place in a particular setting. In Abidjan, Cote d’Ivoire for instance, the cumulative rate of mother-to-child transmission of HIV was ascertained among 250 mother-baby pairs seen between August 2003 and June 2005 [19]. The HIV exposed infants in their study received post exposure prophylaxis and were followed up till their HIV status could be determined. The mothers were divided into two cohorts of those that received HAART (143) and those that had only short course anti-retroviral prophylactic regimen (107). At 12 months, 5.7% (95% CI 2.5%–9.0%) of the infants were diagnosed as HIV-infected: 3.3% (95% CI 0.0%–6.9%) in the HAART group and 7.5% (95% CI 2.8%–12.3%) in the short course ARV for PMTCT group. This cumulative transmission rate of 5.7% is slightly higher than the 3.9% (95% CI 1.1%- 6.7%) cumulative rate documented in this study. This can partly be explained by the fact that all the mothers in this current study received HAART compared to only a proportion from the Abidjan study. In fact, among those that received HAART in the Abidjan study, only 3.3% transmission rate was recorded and this was comparable to the 3.9% documented in the current study. Although the WHO recommends that HAART be given to HIV positive women who need it for their own health in economically-developing countries, several studies have demonstrated the superiority of HAART over short course anti-retroviral regimens in preventing mother-to-child transmission of HIV [20-22]. In the landmark, Pediatric AIDS Clinical Trials Group Protocol 076 Study (PACTG 076), 8.3% in the zidovudine group and 25.5% in the placebo group were HIV infected [21]. The 8.3% HIV transmission rate in the zidovudine group of the PACTG 076 study group differs from the 3.9% documented in this current study [21]. The lower transmission rate in this study may once again highlight the superiority of HAART over monotherapy in reducing rate of peri-natal HIV transmission. In a South African study, the overall rate of MTCT among women receiving HAART was 4.9% [22]. This rate was however lower among the children of women that became pregnant on HAART (0.7%) compared to those who initiated HAART during pregnancy (5.7%) [22].

This cumulative transmission rate of 3.9% in this study very sharply contrasts with another study by Mukhtar-Yola et al. [23] at Kano, North-east Nigeria. Their observational study was also retrospective in nature involving two groups of mother-baby pairs (PMTCT and non PMTCT groups). They found that as high as 25% transmission rate among the PMTCT group and even higher (53.7%) among the non PMTCT group. This may not be surprising as only 18.1% of the mothers in their PMTCT group were on HAART and there was a higher rate of mixed feeding compared with only 7.9% rate of mixed feeding in this study.

Mode of delivery, choice of post exposure prophylactic ARVs for the infants, and infant feeding choice did not have a statistically significant influence on the transmission rate of HIV in this study. Interestingly too, infant feeding choices was not a statistically significant predictors of transmission rate in the Abidjan study [19]. However, the influence of these variables (mode delivery, infant’s ARV prophylaxis and feeding choice) have to be cautiously interpreted in this study as the majority of the babies were delivered per vaginal (87.1%), had SdNVP plus 6 weeks Zidovudine (82.6%) and were on exclusive breast milk substitute for 6 weeks (79.8%). This leaves very few controls to draw reasonable conclusions from these variables. Curiously, all the 7 infected babies were delivered outside UNTH. This may be due to the fact that delivery practices at such places are suboptimal and re-inforces the need to train health workers in best practices. This calls for further scrutiny of the role of place of delivery in the transmission of HIV infection especially with respect to safe blood handling and use of sterilized instruments.

Only 2 out of the 7 infections in this study were recorded among the infants that had some form of breast feeding. This can be explained by the fact that most of the babies (79.8%) in this study received exclusive formula feeding [provided as support by the Government of Nigeria (GoN)] while only 7.9% had mixed feeding. This underscores the importance of exclusive formula feeding in reducing MTCT of HIV. While being associated with reduced HIV transmission in exposed infants, exclusive formula feeding in developing countries is still at huge costs though. These include procuring infant formula, increased rate of common childhood illnesses like diarrhea, respiratory illnesses as well as increased mortality. We therefore, theorise that the bulk of the HIV infections in this study may have happened “in utero” or during delivery. While all the 128 women for whom ante-natal history data were available received HAART, there is the possibility of non-adherence to the anti-retroviral medications during pregnancy by some of the mothers, late presentations, advanced maternal disease, opportunistic infections and severe immune-suppression. These variables were not explored in this review. Additionally, the details of labor and delivery including known risk factors such as prolonged rupture of membrane, episiotomies and instrumental deliveries were not documented in this review.

## Conclusions

This retrospective observational study shows a cumulative 3.9% rate of MTCT of HIV by 18 months of age in Enugu. This may be attributed to a well-established multi-disciplinary team approach between the Obstetric and Pediatric Departments of UNTH Enugu in running the PMTCT program. Commencing infant feeding counselling during the ANC and reinforcing that throughout the duration of ANC and exposed infant follow up may have helped keep the rate of mixed feeding low. Only 7 children were positive, therefore this study lacks statistical power to evaluate the role of risk factors such as maternal HIV disease, ARV prophylaxis, mode of delivery and infant feeding. We therefore, recommend a prospective study to evaluate the role of these risk factors in the MTCT of HIV.

## Methods

### Study population

The HIV exposed infants’ clinic was established at University of Nigeria Teaching Hospital, Enugu in March 2006. It is a weekly clinic created to cater for the specific health needs of HIV exposed children and provide close monitoring that ensures prompt referral of HIV infected children for treatment. The Pediatric HIV clinic is being supported by the AIDS Prevention In Nigeria-Harvard University project through the United States Presidential Emergency Fund for AIDS Relief (PEPFAR). It has a strong linkage with the Obstetrics unit of the hospital which ensures timely referral of all exposed infants delivered in the hospital to the clinic. All HIV positive women seen at UNTH, Enugu are placed on anti-retroviral drugs according to the national guidelines. Such women are provided with infant feeding counselling during HIV post-test counselling. Infant feeding counselling is reinforced throughout the Ante Natal Care (ANC) and exposed infant follow up clinic. The infant feeding choices available at the time were exclusive breastfeeding for short duration (3-6 months) or exclusive replacement feeding using breast milk substitutes. Complementary feeding is then started comprising mainly of local pap gruel made from corn, variedly fortified with soya beans, infant formula, ground paste, crayfish and other locally-available products. The HIV Exposed clinic also receives referrals from health facilities within and outside Enugu metropolis. The babies are started on 2 mg/kg of SdNVP within 72 hours as applicable, then daily 4 mg/kg of suspension Zidovudine for 6 weeks. Cotrimoxazole is started at 6 weeks. The children are followed up every two weeks for the first 6 weeks and then monthly till they attain the age of 18 months. Growth is monitored and infant feeding counselling is provided at each visit. The immunization status is also checked.

Antibody testing alone was used to test for HIV infection in 48 children at 18 months of age who were followed up and discharged before DNA PCR became available. None of these 48 children was breastfed beyond 6 months of age. With the availability of DNA PCR in March 2008, all our remaining clients below 18 months were offered DNA PCR and antibody testing subsequently at 18 months. For babies that were breastfed, repeat DNA PCR was done 6 weeks after cessation of breastfeeding. There was no discordance between the DNA PCR results and the subsequent antibody test results that was done at 18 months. Although DNA PCR is now offered routinely at six weeks, the exposed babies are still followed up irrespective of the DNA PCR result till they are 18 months when antibody testing is also offered.

### Data collection

This was a retrospective study. The case notes/records of all enrolled HIV exposed infants attending the clinic within a 30 month period from March 2006 to Sept 2008 were critically reviewed. Data extracted included gender, birth weight, use of pre delivery ARV in the mother, mode of delivery, infant feeding option; DNA PCR results where available and antibody test result at 18 months of age.

### Data analysis

Retrieved data were validated and analyzed using the Statistical Package for Social Sciences (SPSS) version 19.0 [24]. Each risk factor was tested for statistical association with the risks of HIV infection by 18 months of age. Chi-squared test was used to test for significant association of categorical variables. Graph Pad Prism version 5.04 [25]. (2010) was used to construct the bar plot with error lines for the different feeding choices. A one way Analysis of Variance (ANOVA) was used to test for significant association of the average weights of the infants with various modes of infant feeding.

The level of statistical significance used was 0.05 and 95% confidence interval reported.

### Ethical consideration

Ethical approval was obtained from the UNTH Research and Ethics Committee.

## Competing interests

The authors declare that they have no competing interests.

## Authors’ contributions

NSI, IJE and ANI conceived the study and participated in its design and coordination. ACU reviewed the data, performed the statistical analysis and drafted the manuscript. ACA and KKI retrieved the date from the Medical Recoords Unit, and participated in the writing of the manuscript. All authors read and approved the final manuscript.
